# Ethnic minority experiences of mental health services in the Netherlands: an exploratory study

**DOI:** 10.1186/s13104-022-06159-0

**Published:** 2022-07-28

**Authors:** Onaedo Ilozumba, Trisha Kosher, Elena Syurina, Ikenna Ebuneyi

**Affiliations:** 1 College of Medical and Dental Sciences, Institute of Applied Health Research, University of Birmingham, Edgbaston, Birmingham, B15 2TT UK; 2 Faculty of Science, Vrije Universiteit, Amsterdam, The Netherlands; 3 ALL Institute, Maynooth University, National University of Ireland Maynooth, Maynooth, Ireland

**Keywords:** Mental health, Mental health services, Migrant health, Ethnic minorities, The Netherlands

## Abstract

**Objective:**

Despite considerable spending on mental health in the Netherlands, access to mental health remains suboptimal, particularly for migrants and ethnic minorities. Addressing the growing mental health service needs requires an understanding of the experiences of all stakeholders, specifically minority populations. In this exploratory study, we sought to understand the perspectives and experience of mental health services by migrants and their provider. An exploratory qualitative study was conducted with 10 participants, five of whom were mental health service providers and the other five were clients who had utilized or currently utilized MHS in the Netherlands.

**Results:**

We identified three themes that explained the experiences of clients and providers of MHS in the Netherlands (i) Perceptions of mental health service utilization (ii) Mismatch between providers (iii) Availability of services. The most significant factor that influenced participants experience was a service provider of a different cultural background. Minority populations accessing mental health services have multiple needs, including an expressed need for cultural understanding. Their experiences of mental health services could be improved for minority populations by addressing the diversity of health providers.

**Supplementary Information:**

The online version contains supplementary material available at 10.1186/s13104-022-06159-0.

## Introduction

Global trends indicate a progressive increase in prevalence of mental disorders with an associated increase in disability. According to the WHO, the global prevalence of mental disorders is about 10.7% and in conflict settings, this is about 22.1% [1]. This heightened prevalence has led to several call for increased spending on Mental, Neurological and Substance use disorders to reduce the mental health treatment gap. In the Netherlands, about one quarter of total health budget is spent on mental health [2]; yet access to mental health services is sometimes suboptimal and remains dependent on factors such as health insurance and social class, income, and immigration status [3]. Migrants and persons from ethnic minorities are at higher risk for development of Mental disorders [4, 5] and higher needs for mental health services (MHS). Migration could be associated with adjustment problems, socio-economic problems that predispose them to both mental health problems and poverty with mental health implications [6]. In the Netherlands, the rates of mental health in migrants are higher than in the native Dutch [3, 8]. Studies suggest that the increased rates of mental disorders in migrants may be on account of reduced access to mental health care, (perceived) social exclusion, misdiagnosis, clinical bias, and psychosocial adversity experienced by migrants and ethnic minorities [3, 9–12]. Understanding the actual experiences of mental health services for ethnic minorities and their healthcare providers can also contribute to the promotion of the commitment of the Netherlands and other European Union states to the ethos of inclusive and equitable healthcare [13]. In this exploratory study, we sought to understand the perspectives and experience of mental health services by migrants and their providers.

## Main text

### Methods

We conducted a qualitative exploratory study with a multi-ethnic population who either had a history of MHS utilization or worked within MHS in the Netherlands. Participants were a convenience sample primarily recruited between April and July 2019 via two channels (i) a study flyer (in English and Dutch) which was distributed via social network (ii) networks of person with mental problems. For inclusion in the study participants had to belong to a minority ethnic group and be conversant with MHS in the Netherlands, either as service users or healthcare providers (Table 1). All study participants were legal residents of the Netherlands with a minority ethnic background.

**Table 1:** Participant characteristics

Participant code	Gender	Country of origin	Patient or provider	Self-reported diagnosis
P_01	Male	Antillean	HCP (MHS support staff)	N/A
P_02	Female	Filipino	Patient	Depression
P_03	Female	Surinamese	Patient	Depression
P_04	Female	Iranian	Patient	Depression
P_05	Female	Antillean and Dutch	HCP	N/A
P_06	Male	Afghan	HCP (GP)	N/A
P_07	Female	Antillean and Dutch	HCP (MHS Manager)	N/A
P_08	Male	Cape Verdean	HCP (MHS nurse specialist)	N/A
P_09	Female	Turkish	Patient	Depression
P_10	Female	Turkish	Patient	Depression and anxiety

In-depth interviews were conducted using a semi-structured interview guide which had been pilot tested with experts in MHS. The interview guide was developed in Dutch for this study and a translated version is available in Additional file 1. Prior to the interview the participants were sent an informed consent which included information about the study and researchers. TK, a female researcher who was trained in qualitative interviewing techniques and a native Dutch speaker and proficient in English Language conducted all interviews alone. Interviews were conducted by phone or at a neutral location of the participants choosing. Interviews lasted for approximately 30 min and were recorded, transcribed, and translated to English from Dutch. Interviews explored participants experiences with MHS, including the duration of their contact with MHS, experiences of interacting with health providers or clients, social support while seeking MHS and barriers and facilitators to MHS access and utilization. TK took field notes during the interviews, and after transcription participants were provided summarised research notes and could provide feedback or corrections.

Thematic content analysis was undertaken by two independent researchers to identify themes consisted with the study objectives. The first stage of analysis involved the open coding of transcripts to identify common codes and preliminary sub-themes and themes. A codebook was developed based on the initial codes and analysis. All transcripts were re-coded using the developed codebook with emergent codes included in the codebook. The final themes were decided upon by the researchers after discussion and reflection on the results.

## Result

### Perceptions of mental health service utilization among ethnic communities

All participants discussed the negative and stigmatized perceptions of mental health. Participants consistently talked about the perception that individuals who seek MHS are “crazy”; This label related to what all participants discussed as a taboo in seeking mental health care among their communities. Taboos were related very strongly to participants disinclination to seek mental health services or their desire to keep their mental health needs secret. One reason given for these perceptions was a lack of knowledge and old-fashioned perceptions about mental health care and institutions.

“Um, yes I think it's still a bit taboo and um just not enough knowledge of it. Or that they think yes psychiatry, psychologist, psychiatrist, that's where you go when you're crazy. And um have a very different view of psychiatry than it actually is.” (Participant 7, Provider).

Some participants also reported differing responses from their immediate families. These experiences varied depending on the existing relationship with parents and the participant’s age. Some participants reported that individuals in their social support network wanted to be supportive but did not understand mental health. Additionally, participants discussed strong cultural norms around not discussing personal issues outside of the home setting.

“My mother was open that I was going, but she was sceptical about it herself. You go to a psychologist if you are crazy in quotation marks, and they have that very much in their head even if you feel a little worse or less comfortable in your skin that you can talk to someone…And you shouldn't discuss your business from home with the outside, that's a big deal.” (Participant 3, Client).

### Mismatch between providers and clients

A common complaint through all interviews was the homogeneity of health practitioners in the Dutch mental health system. All participants shared the views or experiences that the Dutch health system comprised mostly of Dutch-speaking practitioners with Dutch backgrounds, this lack of diversity affected the perception of care. One major way in which this influenced the perception of care was regarding language. Language mismatches were sometimes due to the client being unable to speak Dutch and the practitioner not speaking the client’s language. However, it was also related to participants feeling unable to properly express themselves and their complex thoughts and emotions in Dutch.

“a Dutch psychiatrist is less able to empathize with an Antillean client, for example. Yes, you see that very often there is and remains a language barrier and if [Dutch] is not your mother tongue, for example, then it is difficult to express yourself in that language.” (Participant 7, Provider).

While the cultural match was important, it also appeared that what was necessary was not a direct match between cultures. However, an individual with some understanding of minority cultures was perceived as being more understanding of the cultural considerations. This is illustrated by a Turkish clients (participants) discussion of her Cape Verdean psychologist.

“.. a Cape Verdean man and in terms of culture he knows quite a lot and I like that too, because yes you don't take me wrong, because I was also just born and raised Dutch, and I am also just Dutch for my feelings. But of course, I have a background of culture and what's just nice, um yes, he understands” (Participant 9, Client).

The third aspect of cultural mismatch was expressed by two participants who began utilizing mental health services as minors. They expressed a frustration as the inability of health care professionals to understand the constraints of their culture, acceptable behaviour and the rules that governed their lives.

“Um, it's not necessarily that she did it consciously or anything… I was 17 at the time so I was with a child psychologist at the time, …you noticed that there were differences in their thinking and my way of thinking. For example, they said you're almost 18 so you can do this and this. And that doesn't quite work in the culture and then she did try to come up with other solutions or some things I thought of that is not entirely realistic in my situation so in that area it sometimes clashed.” (Participant 3, Client).

### Availability of services

In relation to the access and availability all clients of mental health services had positive experiences and did not experience any barriers with regards to gatekeepers or finances. Clients and practitioners did not discuss any ethnic or sociodemographic health system barriers to accessing mental health services. However, a commonly discussed problem was the presence of waiting lists, while the client participants themselves had not experiences this, they all knew of individuals who had experienced significant delays in receiving care. This was collaborated by health providers who discussed long waiting lists, limited mental health service providers, insufficient training of new mental health workers and limited finance.

“Very long waiting times, uh from locker to wall. And the larger the mental health institution the worse. A lot of expiration days, then you hear from patients of mine had to wait three four months before I could see the psychologist. After an intake, a next intake comes and then they get a note of maternity leave, check it out. I will be here in 6 months and my colleague will guide you further and that colleague is sick, and you name it. That kind of crap.” (Participant 6, Provider).

In such a system with long waiting lists, participants discussed the importance of understanding the Dutch healthcare system, including insurances and referral system. Some participants also discussed that non-ethnic Dutch population often had limited knowledge related to navigating the health system which could also affect their experiences of seeking care and ultimately mental health outcomes.

## Discussion

In this study a lack of diversity in the mental health service was found to significantly influence the mental health experiences of ethnic minorities in the Netherlands. Participants discussed language as a common barrier to the experience of mental health services, like the findings of other studies [14–16]. However, unlike studies conducted among minorities in the United States the study participants all had a working knowledge of Dutch. They were able to read and communicate in the language but felt limited in expressing complex emotions and thoughts in a non-native language as well as differences in cultural habits and customs.

We found that misconceptions existed among minority ethnic populations about the nature of mental health services, it is commonly assumed that accessing mental health services would lead to a loss of freedoms and ill-treatment. This was linked to the description of individuals with mental health illnesses as crazy. The stigmatization of mental health problems and the need for MHS services often resulted in secrecy among MHS service users. However, some other studies which have discussed culture in terms of supernatural believes, racism, post-traumatic stress and trust our study highlighted more of participants sense of not being understood [3, 9]. Their way of life and expression were not actively integrated into their care pathways. In our study, adolescents reported a mismatch between the advice they received from health service providers and the expectations of their parents and social networks. This was particularly true of adolescents who received advice that were not suitable for their home situations from health service providers. The WHO mental health promotion and mental health care in migrants and refugees recommends offering culturally appropriate mental health services to achieve better health outcomes [17].

In our study migrants and ethnic minorities with less severe mental health concerns did not face overt systematic discrimination or barriers in accessing care. Rather their experience of care was most influenced by the lack of diversity among MHS providers in the Netherlands. The recommendation given by all participants was a need for more MHS providers, including a greater emphasis on diverse ethnic backgrounds. According to Alsan et al., diversity in healthcare workforce has positive effects on health service utilization and outcomes [18]. Given the multicultural increase in migration and mental health needs among ethnic minority population understanding the MHS experiences is of greater importance. Improved health cannot be achieved with addressing MHS and without ensuring that all members of the society have equitable access to high quality MHS.

## Limitations

In this study all clients reported having depression and or anxiety, unsurprising given the prevalence of depressed mood among ethnic minority populations in the Netherlands [20]. However, this is also a limitation of the study as it could be that individuals with more severe forms of mental health disorders, those requiring institutional care might have different experiences and needs [21].

## Supplementary Information

**Additional file 1.** Topic guide for participant interviews.

## Data Availability

Transco analysed during the current study are available from the corresponding author on reasonable request, taking into consideration the adherence to ethical approval obtained for this study, and adherence to confidentiality.

## Abbreviation

MHS: Mental Health Services
