# Correction: Ethnic minority experiences of mental health services in the Netherlands: an exploratory study

**DOI:** 10.1186/s13104-022-06168-z

**Published:** 2022-08-09

**Authors:** Onaedo Ilozumba, Trisha Kosher, Elena Syurina, Ikenna Ebuenyi

**Affiliations:** 1 College of Medical and Dental Sciences, Institute of Applied Health Research, University of Birmingham, Edgbaston, Birmingham, B15 2TT UK; 2 Faculty of Science, Vrije Universiteit, Amsterdam, The Netherlands; 3 ALL Institute, Maynooth University, National University of Ireland Maynooth, Maynooth, Ireland

## Correction: BMC Research Notes (2022) 15:266 10.1186/s13104-022-06159-0

Following the publication of the original article [1], the authors reported an error in the last author's surname:

- The name "Ikenna Ebuneyi" is incorrect.
- The correct name is "Ikenna Ebuenyi".

The correct name is captured both in the author list of this Correction and in that of the original article [1], which has already been revised.
