# Supplementary material for: Ethnic minority experiences of mental health services in the Netherlands: an exploratory study
Source: BMC Res Notes. 2022 Jul 28;15:266. doi: 10.1186/s13104-022-06159-0 (PMC9331111; doi:10.1186/s13104-022-06159-0)
Supplement: Supplementary file 1 — Additional file 1. Topic guide for participant interviews. [file 13104_2022_6159_MOESM1_ESM.docx]

**Topic Guide Patients**

Opening questions

- Are you a migrant according to the general definition?
- How are you familiar with mental health services?
- Could you tell me which MHC services you are using or have used? (e.g. psychologist, institution etc.)
- When did you first come in contact with mental health services?

Introductory questions

- What is your general opinion about mental health care in the Netherlands?
- When you first used a mental health service; did you have certain expectations?
- If yes? What were these expectations and how would you describe them?
- If no? Could you explain why you did not have any expectations?

Transition questions

- Have you ever considered to abstain from using mental health services?
- If yes, what was your main reason for this abstention?

Main questions

*Intrapersonal level*

- What is your personal attitude towards mental health services and professionals?
- If you keep your situation in mind, how did you experience mental health services and professionals?
- Have you ever experienced negative service by GGZ professionals because of your ethnic background?
- If yes? what did you notice in that case?
- Have you ever experienced positive service by GGZ professionals because of your ethnic background?
- If yes? what did you notice in that case?

*Interpersonal level*

- How would you describe the influence of your social networks (such as school, work, community in which you live) in your perspective on the use of mental health care?
- How has your background and environment shaped your perspective on mental health care?
- Given your work or school environment. Have these factors ever prevented you from using mental health services?
- If yes, how did you noticed that?
- How did your family (background) play a role in the use mental health care? (or the abstention thereof?)

*Institutional level*

- Were there regulations that prevented you from receiving appropriate mental health care?
- If yes, how did you noticed this?
- How did you deal with this?
- What is your perspective on the role of the government in mental health services?

*Community level*

- How would you describe the perspective of Dutch culture towards mental health care?
- How would cultural norms and beliefs (of this country) affect an individual to receive mental health care?

*Public Policy level*

- If mental health care is missing something according to your experiences and perspectives. What is missing?
- What can the government do to improve services?

Final questions

- Is there anything you want to add about your experience / perspective on mental health services that has not yet been mentioned?

**Topic Guide MHC professionals**

Opening questions

- Can you describe your profession and function?
- How would you describe the demographics of your patients? (for example, does the majority of patients have a non-Western background?)
- How can you explain this (lack of) diversity in patients?

Introductory questions

- What are your views on mental health care in the Netherlands?
- Do you notice that there is a difference in the use of mental health care by Dutch patients compared to patients with a non-Western background?
- If yes, how do you notice this?
- What are the main reasons for this difference?
- Do you notice that Dutch patients have different experiences with regard to mental health care compared to non-Western immigrants?
- If yes? What are the subjects on which they express their dissatisfaction?
- If not? what is the explanation for this?

Transition questions

- Patients may deny that they need mental health care. How would you explain the main reasons for avoiding MHC?

Main questions

*Intrapersonal level*

- What are the general thoughts / opinions / attitudes of non-western patients / clients regarding mental health care in the Netherlands?

*Interpersonal level*

- Do you notice that the use of mental health care is influenced by the social networks of the non-western patient, such as school, work, community in which they live?
- If yes, How?
- How does the culture of the patient play a role in obtaining mental health care?
- How can you describe this phenomenon?

*Institutional level*

- Are there regulations or procedures in the mental health care that keep the patient from getting the proper care?
- If yes? Could you describe them?

*Community level*

- How would you describe the Dutch culture (norms and values) with regards to mental health care?
- How would the cultural norms and beliefs of the non-western patient affect the use of mental health care?

*Public policy level*

- What could the government do to improve mental health care services?
- How can mental health care be improved in the field of cultural inclusiveness?
- How can the experience of ethnic minorities be improved?

Final questions

- Is there anything you want to add about your experience / perspective on mental health services that has not yet been mentioned?
